# Evaluation of Explicit Motor Timing Ability in Young Tennis Players

**DOI:** 10.3389/fpsyg.2021.687302

**Published:** 2021-06-24

**Authors:** Ambra Bisio, Emanuela Faelli, Elisa Pelosin, Gloria Carrara, Vittoria Ferrando, Laura Avanzino, Piero Ruggeri

**Affiliations:** ^1^Section of Human Physiology, Department of Experimental Medicine, University of Genoa, Genoa, Italy; ^2^Centro Polifunzionale di Scienze Motorie, University of Genoa, Genoa, Italy; ^3^Department of Neuroscience, Rehabilitation, Ophthalmology, Genetics, Maternal and Child Health, University of Genoa, Genoa, Italy; ^4^Istituto di Ricovero e Cura a Carattere Scientifico Ospedale Policlinico San Martino, Genoa, Italy

**Keywords:** tennis, sport neuroscience, motor performance, motor expertise, timing

## Abstract

A crucial ability for athletes playing sports that involve coincidence timing actions is the motor timing ability. The efficiency of perceptual and motor processes underlying the motor timing ability has been related to the motor experience gained in interceptive sports, such as tennis. In the present study, the motor timing ability in young tennis players (TP) and age-matched control participants (CTRL) was compared by means of a synchronization paradigm. Participants were asked to perform finger-opposition movements in synch to a metronome beating 0.5 and 2 Hz in (1) a bimanual coordination test, which compared the performance of the dominant hand with that of the contralateral hand, and (2) a movement lateralization test, which compared the motor performance of the dominant hand during single-hand and bimanual tasks (BTs). The motor performance was evaluated through movement strategy [defined by touch duration (TD), inter-tapping interval (ITI), and movement frequency] and movement accuracy (temporal accuracy defined by the synchronization error and spatial accuracy defined by the percentage of correct touches—%CORR_SEQ). Results showed that motor expertise significantly influences movement strategy in the bimanual coordination test; TD of TP was significantly higher than those of CTRL, specifically at 0.5 Hz. Furthermore, overall ITI values of TP were lower than those of CTRL. Lastly, in the movement lateralization test, the %CORR_SEQ executed with the right dominant hand by TP in the BT was significantly higher than those of CTRL. A discussion about the role of motor expertise in the timing ability and the related neurophysiological adaptations is provided.

## Introduction

Motor timing is a key functional domain that plays a crucial role in making our actions efficient and appropriate to the context (Bueti et al., 2008). It can thus be considered a prerequisite for appropriate reactive and proactive behavior. In case of coincidence timing actions, such as catching or hitting balls, the subject must tune actions with the approach of moving objects in order to intercept them. The accuracy of this response is likely to depend on the perceptual and motor processes, including timing information, which link the sensory input to the motor output (Abernethy and Burgess-Limerick, 1992; Tresilian, 1995). The efficiency of these processes has been related to the motor experience gained, for instance, in interceptive sports such as table tennis (Ripoll and Latiri, 1997) and tennis (Benguigui and Ripoll, 1998). These sports involve serial skills that, during long rallies, include repetitive sequential movements, which are marked by salient events (i.e., ball interception) interleaved by pauses (i.e., the period between interceptions). Motor control processes involved in this kind of task require an explicit event-related temporal representation of the target interval and were described as event timing by Ivry et al. (2002). One may hypothesize that the timing ability gained in interceptive sports might not be specific for the coincident timing actions, but concerns all the temporal processes involved in tasks requiring an explicit representation of a target interval as suggested by Zelaznik et al. (2002).

Explicit motor timing ability can be evaluated by executing motor tasks, in which participants make explicit use of temporal information (e.g., estimates of the duration of stimuli or intervals between stimuli) to represent a time interval through a motor action (Coull and Nobre, 2008). One of the most commonly adopted explicit timing task is the synchronization paradigm, which requires participants to perform sequential movements in synchrony with a train of tones separated by a constant interstimulus interval (ISI) (Bonzano et al., 2008, 2013a,b, 2017; Avanzino et al., 2013a,b; Pardini et al., 2013; Martino et al., 2016, 2017, 2019; Signori et al., 2017, 2020). In that condition, the acoustic signal might define the temporal goal, namely the salient event used by motor control processes to guide the motor response (Zelaznik et al., 2002, 2005).

The synchronization paradigm was proposed at both sub-second and supra-second ISIs in order to test the involvement of different neural networks. MRI investigations revealed activity of sensorimotor areas, supplementary motor area, and cerebellum in case of sub-second ISI (e.g., 500 ms, metronome frequency: 2 Hz), associated with the so-called automatic timing system (Lewis and Miall, 2003; Breska and Ivry, 2016). The “cognitively controlled timing system” was instead proposed to regulate timing events at supra-second ISI (e.g., 2,000 ms, metronome frequency: 0.5 Hz) when, together with basal ganglia, prefrontal and parietal associative areas are involved (Lewis and Miall, 2003).

In this study, the motor performance in tasks requiring explicit motor timing abilities was explored by means of a synchronization paradigm in young tennis players (TP) and age-matched control participants (CTRL). The first aim was to investigate whether expertise gained in a highly asymmetrical sport like tennis (Ducher et al., 2005; Lucki and Nicolay, 2007) might influence upper-limb motor performance in general, as a result of a gained high-level timing ability acting on response planning irrespective to the effector of movement, or specifically for the dominant side. This was done by comparing movement strategy and accuracy of the motor performance of the dominant hand with that of the contralateral hand during the execution of a bimanual task (BT) consisting in metronome-paced finger-opposition movement (bimanual coordination test). The second aim was to assess whether expertise influenced the level of asymmetry evaluated on the dominant hand during single-hand and bimanual motor performance. To this purpose, the motor performance during a single-hand finger-opposition movement task was compared to that acquired during the same task executed with both hands, both of them in synch with a metronome (movement lateralization test). Since performance on the synchronization test is largely dependent on the duration of the ISI due to the involvement of different neural networks (Lewis and Miall, 2003), the metronome rate was set at both 2 Hz (i.e., sub-second ISI: 500 ms, a time interval closely related to the individual spontaneous movement tempo; Bove et al., 2009) and 0.5 Hz (i.e., supra-second ISI: 2,000 ms).

## Methods

### Participants

About 20 TP (seven females and 13 males; mean age ± SD = 13.75 ± 2.47 years, range: 10–18 years; mean years of practice ± SD = 4.80 ± 2.02, range 1–9 years; mean hours of training per week ± SD = 2.35 ± 0.61) were recruited from local tennis teams in Genoa, Italy. All TP performed the backhand stroke with two hands. About 22 healthy CTRL, who never played tennis, were recruited from local schools in the same geographical area (nine females and 13 males; mean age ± SD, 14.32 ± 2.42 years; range, 11–18 years). CTRL were either sedentary (*n* = 7) or practiced sports other than tennis (water polo *n* = 2; football *n* = 3; basketball *n* = 1; athletics *n* = 5; dance *n* = 2; rugby *n* = 1; and horse riding *n* = 1). Participants were consistent right-handers according to the Edinburgh handedness inventory (Oldfield, 1971). A written informed consent was obtained from all participants and legal guardians before data collection. The study was approved by the ethical committee of the University of Genoa (Comitato Etico per la Ricerca di Ateneo, *n*° 2021.31) and was conducted in accordance with the Declaration of Helsinki.

### Experimental Paradigm

**Figure 1:**
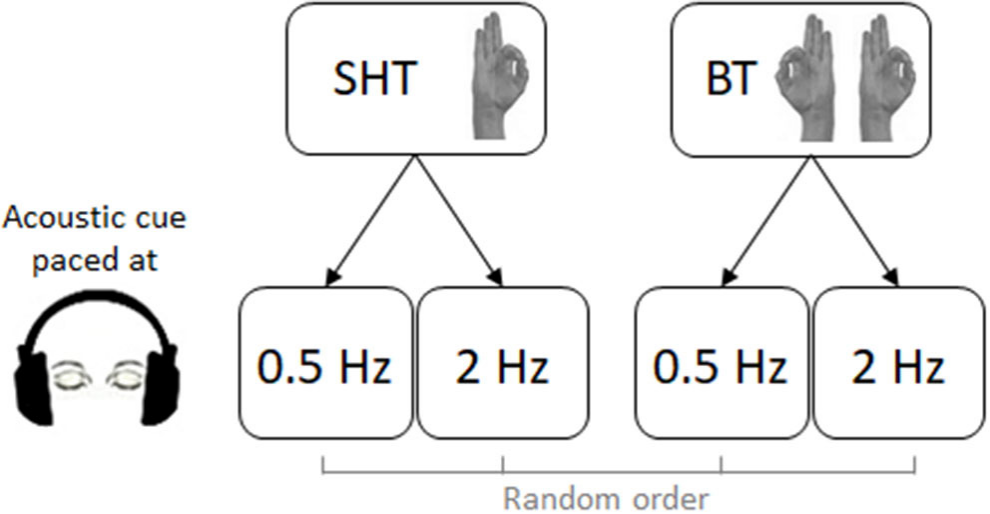
Experimental paradigm. Subjects were required to perform a finger-opposition movement sequence (opposition of the thumb to the index, medium, ring, and little fingers) in synchrony with a metronome at 0.5 and 2 Hz, with the right hand only (single hand task—SHT) or with both hands (bimanual task—BT), for a total duration of 45 s. The four experimental conditions were executed in a random order.

Subjects were seated in a comfortable chair in a quiet room. They wore a sensor-engineered glove (Glove Analyzer System, eTT s.r.l., Genova, Italy) on both hands. Subjects were instructed to perform a sequence of finger opposition movements (opposition of thumb to index, medium, ring, and little fingers) following an acoustic cue paced at 0.5 and 2 Hz. Participants performed this task with the right hand (RH) only [single-hand task (SHT)] or with both hands simultaneously (BT) in synchronization with the metronome for a total duration of 45 s, followed by a 1-min rest. In summary, participants performed four tasks in a random order: SHT at 0.5 Hz, SHT at 2 Hz, BT at 0.5 Hz, and BT at 2 Hz (Figure 1). The comparison of RH and left-hand (LH) performance during the BT allows evaluating bimanual coordination (afterward named bimanual coordination test). By comparing RH performance in SHT and BT, it was possible to test whether motor experience in tennis influences movement lateralization (afterward named movement lateralization test).

### Data Analysis

The outcome parameters allowed the description of the motor strategy and the motor performance accuracy in the bimanual coordination test and in the movement lateralization test.

In particular, the following kinematic parameters were computed to investigate the motor strategy: (1) touch duration (TD; contact time between the thumb and another finger, ms); (2) inter-tapping interval (ITI; the time interval between the end of a thumb-to-finger contact and the beginning of the subsequent contact in the finger motor sequence, ms); and (3) movement frequency (FREQ; how many thumb-to-finger contacts occurred in 1 s, computed as 1/(TD + ITI), Hz).

Performance accuracy was described using spatial and temporal accuracy. To describe spatial accuracy of the motor performance, the number of correct movements, expressed as a percentage of the total sequence number—%CORR_SEQ—was considered. Temporal accuracy was defined as the ability of participants to move in sync to the metronome and was described with the synchronization error (SYNCH_ERR, ms), computed as ISI – (TD + ITI). This parameter provides a direct measure of the magnitude of the error in reproducing the corresponding time interval (Hary and Moore, 1987; Avanzino et al., 2013b; Martino et al., 2019). Positive SYNCH_ERR values indicated that the motor response was in advance with respect to the metronome beat, whereas negative values indicated a delayed answer.

The Shapiro–Wilk tests confirmed that the parameters were normally distributed.

In order to evaluate bimanual coordination, repeated-measure ANOVAs with RATE (two levels, 0.5 and 2) and HAND (two levels, RH and LH), as within-subject factors, and GROUP (two levels, TP and CTRL) as between-subjects factor were applied on TD, ITI, FREQ, %CORR_SEQ, and SYNCH_ERR.

Movement lateralization was statistically investigated by means of repeated-measures ANOVA on the same parameters as before, but considering the RH only, with RATE (two levels, 0.5 and 2) and TASK (two levels, SHT and BT), as within-subject factors, and GROUP (two levels, TP and CTRL) as between-subjects factor.

The correlation analysis between movement kinematics and motor performance accuracy of participants with age, experience in tennis (years of practice), and hours of training per week was performed.

The Bonferroni *post-hoc* test was used to evaluate significant interactions. Significance for all procedures was set at a level of 0.05. Data are presented as mean ± SEM. The statistical analyses were performed using the SPSS software (SPSS, Inc., Chicago, IL, USA).

## Results

### Bimanual Coordination Test

#### Movement Strategy

Repeated measure ANOVA on TD showed significant main effects of RATE [*F*_(1, 40)_ = 71.91, *p* = 0.000, η^2^ = 0.64], indicating that TD at 0.5 Hz (318.65 ± 16.47 ms) was significantly longer than at 2 Hz (179.65 ± 5.26 ms). TD was significantly longer in TP (268.75 ± 13.13 ms) than in CTRL (229.54 ± 12.52 ms) as indicated by the significant main effect of GROUP [*F*_(1, 40)_ = 4.67, *p* = 0.037, η^2^ = 0.11]. Lastly, the significant RATE^*^GROUP interaction [*F*_(1, 40)_ = 4.20, *p* = 0.047, η^2^ = 0.10] showed that TD of TP was longer than that of CTRL only when the metronome was set at 0.5 Hz (*p* = 0.03).

The ITI value of TP (967.82 ± 15.46 ms) was significantly lower than that measured in CTRL (1013.42 ± 14.74 ms) [GROUP: *F*_(1, 40)_ = 4.56, *p* = 0.039]. Moreover, the ITI value at 0.5 Hz (1663.97 ± 21.12 ms) was significantly higher than at 2 Hz (317.28 ± 8.02 ms) [RATE: *F*_(1, 40)_ = 3211.72, *p* = 0.000, η^2^ = 0.99].

**Figure 2:**
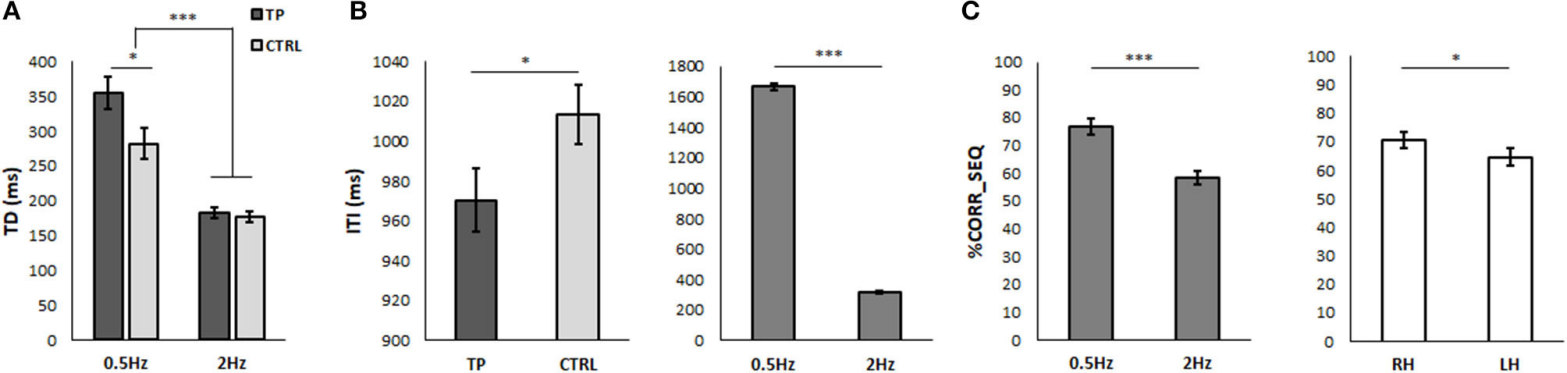
Movement strategy **(A,B)** and performance accuracy **(C)** in the bimanual coordination test. Mean values of touch duration [TD, **(A)**] and inter-tapping interval [ITI, **(B)**] of tennis players (TP, dark gray) and control participants (CTRL, light gray), when the metronome was set at 0.5 and 2 Hz. **(C)** Representation of mean values of the number of correct movements expressed as a percentage of the total sequence number (%CORR_SEQ) when the metronome was set at 0.5 and 2 Hz **(A)** and when the task was executed with the right (RH) and left (LH) hand **(B)**. Error bars indicate SEM; *p < 0.05 and ***p < 0.001, respectively.

Results are shown in Figures 2A,B.

The results of the statistical analysis on FREQ showed a significant main effect of RATE [*F*_(1, 40)_ = 3923.60, *p* < 0.0001, η^2^ = 0.99], indicating that when the task was performed at 0.5 Hz, the movement frequency of participants was significantly lower than when the metronome was set at 2 Hz (metronome 0.5 Hz: 0.51 ± 0.003 Hz; metronome 2 Hz: 2.02 ± 0.02 Hz). No significant main effect of GROUP and no significant interaction involving the GROUP factor were found.

#### Performance Accuracy

The statistical analysis on %CORR_SEQ (Figure 2C) showed that this value was significantly higher when the task was performed at 0.5 Hz (76.78 ± 2.86) than at 2 Hz (58.50 ± 2.43) [RATE: *F*_(1, 40)_ = 28.27, *p* < 0.001, η^2^ = 0.41], and when the task was executed with the RH (70.63 ± 2.73) than with the LH (64.65 ± 3.11) [HAND: *F*_(1, 40)_ = 5.89, *p* = 0.020, η^2^ = 0.13]. No significant difference appeared between the groups, and no significant effects were found in %CORR_SEQ bimanual index.

No significant effect of RATE, HAND, and GROUP and no significant interactions were revealed by the results of the statistical analysis on SYNCH_ERR on both TP (0.5 Hz: RH 25.01 ± 13.32 ms, LH 28.75 ± 12.72 ms; 2 Hz: RH −6.32 ± 9.54 ms, LH 6.27 ± 6.94 ms) and CTRL (0.5 Hz: RH −1.82 ± 12.70 ms, LH −3.96 ± 12.13 ms; 2 Hz: RH 5.60 ± 9.10 ms, LH 6.77 ± 6.61 ms) groups.

### Movement Lateralization Test

#### Movement Strategy

**Figure 3:**
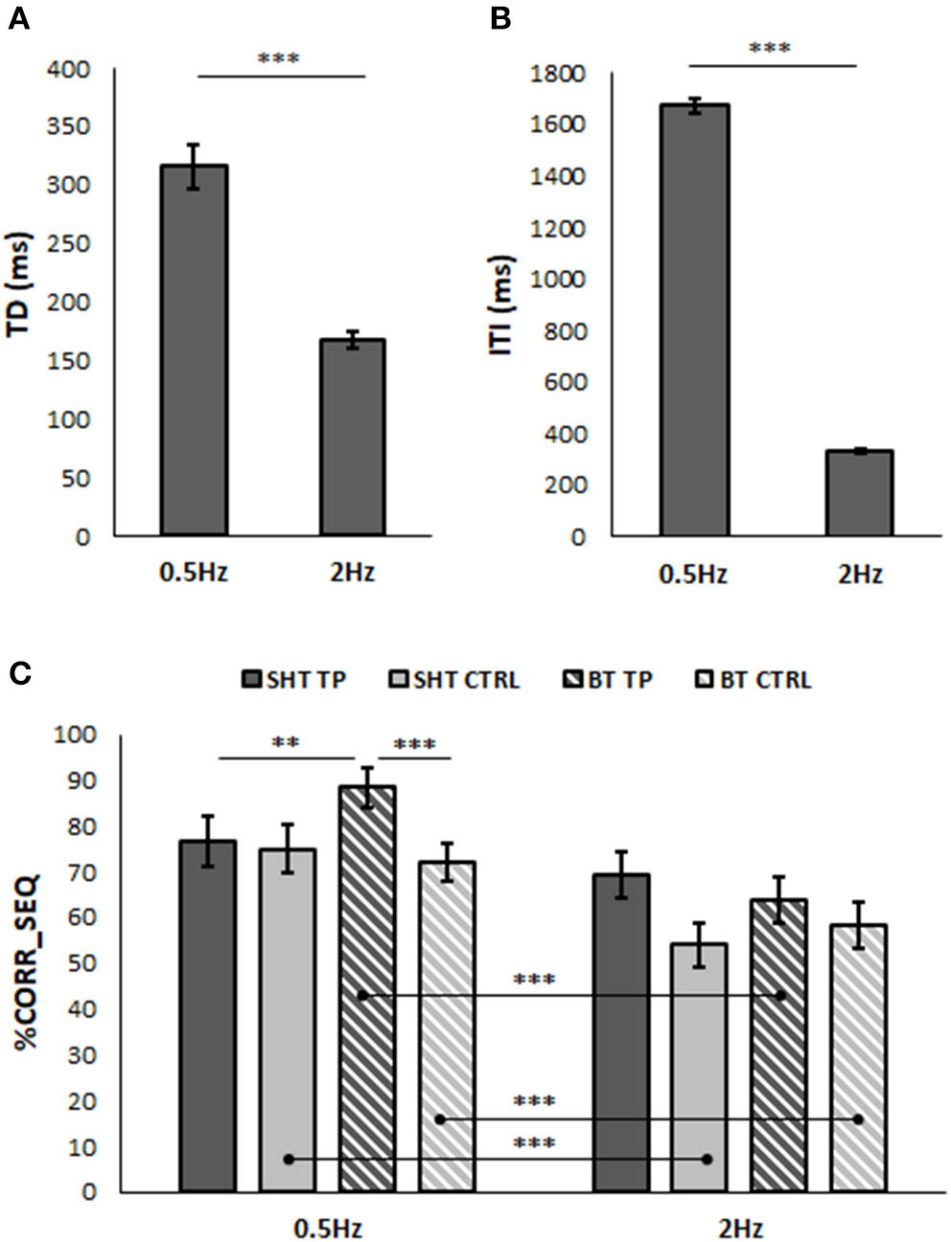
Movement strategy **(A,B)** and performance accuracy **(C)** in the movement lateralization test. Mean values of touch duration [TD, **(A)**] and inter-tapping interval [ITI, **(B)**] when the metronome was set at 0.5 and 2 Hz. **(C)** Representation of mean values of the number of correct movements expressed as a percentage of the total sequence number (%CORR_SEQ) when the metronome was set at 0.5 and 2 Hz during single-hand task (SHT) and bimanual task (BT) by tennis players (TP, dark gray) and control participants (CTRL, light gray). Error bars indicate SEM; **p < 0.01, ***p < 0.001.

Analyses on movement kinematic parameters showed significantly higher TD and ITI values in correspondence of 0.5 Hz rate (TD: 315.55 ± 19.56 ms; ITI: 1671.99 ± 25.45 ms) than when the metronome was set at 2 Hz (TD: 167.69 ± 6.37 ms; ITI: 330.68 ± 8.96 ms) [TD: *F*_(1, 40)_ = 62.27, *p* < 0.001, η^2^ = 0.61; ITI: *F*_(1, 40)_ = 2569.33, *p* < 0.001, η^2^ = 0.99]. No significant main effect of GROUP and no interactions involving GROUP were found. Results are displayed in Figures 3A,B.

Participants fulfilled the task and moved in synch to the metronome rate. Indeed, the result of the statistical analysis showed that the movement frequency of participants when the task was performed at 0.5 Hz (0.5 ± 0.003 Hz) was significantly lower than when the task was performed at 2 Hz (2.02 ± 0.02 Hz) [FREQ: *F*_(1, 40)_ = 4381.36, *p* < 0.001, η^2^ = 0.99]. No significant main effect of GROUP and no significant interaction involving the GROUP factor were found.

#### Performance Accuracy

Concerning %CORR_SEQ (Figure 3C), ANOVA indicated a significant main effect of RATE [*F*_(1, 40)_ = 26.84, *p* < 0.001, η^2^ = 0.40] and a significant RATE^*^GROUP^*^TASK interaction [*F*_(1, 40)_ = 9.14, *p* = 0.004, η^2^ = 0.19]. The Bonferroni *post-hoc* comparisons on the TP dataset showed significantly higher %CORR_SEQ values in BT at 0.5 Hz (88.33 ± 4.21) than at 2 Hz (63.91 ± 5.15) (*p* < 0.001), whereas no differences appeared in SHT (0.5 Hz: 76.67 ± 5.66; 2 Hz: 69.13 ± 5.03). In the CTRL dataset, %CORR_SEQ values at 0.5 Hz were significantly higher than at 2 Hz in both SHT (0.5 Hz: 75.00 ± 5.40; 2 Hz: 54.15 ± 4.80; *p* = 0.001) and BT (0.5 Hz: 71.97 ± 4.01; 2 Hz: 58.30 ± 4.91; *p* = 0.008). Furthermore, in the TP group at 0.5 Hz, %CORR_SEQ was significantly higher in BT than in the SHT group (*p* = 0.01). Lastly, significant differences appeared also between groups. In particular, at 0.5 Hz during BT, %CORR_SEQ of TP group was significantly higher than that of CTRL group (*p* = 0.008).

No significant effect of RATE, HAND, and GROUP and no significant interactions were found by the statistical analysis on SYNCH_ERR on both TP (0.5 Hz: SHT 14.28 ± 11.22 ms, BT 25.01 ± 13.32 ms; 2 Hz: SHT −2.77 ± 6.49 ms, BT −6.32 ± 9.54 ms) and CTRL (0.5 Hz: SHT 18.65 ± 10.70 ms, BT −1.82 ± 12.70 ms; 2 Hz: SHT 10.05 ± 6.19 ms, BT 5.60 ± 9.10 ms) groups.

### Correlations

Movement kinematics and motor performance accuracy of TP in both tests did not significantly correlate either with age, experience in tennis playing (years of practice), or with the hours of training per week (*p*-value always >0.05).

## Discussion

The aim of this study was to evaluate the impact that the motor expertise gained in a highly asymmetrical sport like tennis has on bimanual coordination and movement lateralization motor timing tasks. Results showed that in the bimanual coordination task, when performed in synch to a metronome set at 0.5 Hz, motor expertise influenced movement strategy; in particular, TD of TP was significantly higher than that of CTRL. Furthermore, ITI values of TP were lower than the ITI values of CTRL. It was also shown that, during movement lateralization task at 0.5 Hz, motor experience had an impact on the motor performance spatial accuracy: namely, the %CORR_SEQ executed with the right dominant hand by TP in BT was significantly higher than that of CTRL. Furthermore, %CORR_SEQ in this condition was significantly higher than that measured when the task was performed by TP with one hand (SHT), and when the task was performed bimanually (BT) at 2 Hz. Finally, no difference emerged between TP and CTRL on temporal accuracy for both tasks and metronome frequencies.

### Motor Expertise Influences Movement Strategy in Bimanual Coordination Test

Differences between groups in the bimanual coordination test appeared in TD and ITI, which are kinematic parameters describing the movement strategy. TD may be considered as the combination of a sensory phase and a motor preparation phase in which the successive movement is planned prior to the execution, whereas ITI is likely to be a pure motor component of the task (Bisio et al., 2015). However, it has to be noted that when the task is performed in sync to the metronome, as in the present case, the main aim of participants is to follow the beat; therefore, both parameters are subjected to the rhythmic abilities (i.e., perceptual and motor abilities).

A growing body of evidence indicates the role and the importance of rhythm in tennis. Having a good rhythmic ability might help TP to obtain harmonious movements (Bourquin, 2003) and to have an efficient synchronization of movements with the external stimulus, which is the ball trajectory (Zachopoulou et al., 2000). Specific trainings aimed at improving rhythm in TP were proposed, underlying the impact that a good rhythmic ability has on the tennis performance (Zachopoulou and Mantis, 2001; Sögüt et al., 2012). Therefore, one might hypothesize that the exposure to a sport that is characterized by serial skills including repetitive sequential movements, which are marked by salient events (i.e., ball interception), may be responsible for the acquisition of the movement strategy observed here. Particularly, TP dedicated a shorter time to pass from one finger to the other (ITI) and consequently a longer time to the contact between fingers (TD) with respect to controls, possibly resulting from the experience in a sport involving coincidence timing actions (e.g., hitting a ball). Indeed, in tennis, and in table tennis, athletes need to react in a fast-moving environment (Padulo et al., 2016). This requires athletes to reduce both reaction and movement times (here included in ITI) and might explain the present difference with nonexperts.

It has to be noted that the difference between TP and CTRL in TD was specific for 0.5 Hz (i.e., slow movement), whereas TD values were comparable between groups when the task was performed at 2 Hz (i.e., fast movement). Previous studies showed that spontaneous movement tempo, namely the individual preferred rhythm produced when freely performing tapping/marching movements, is set around 2 Hz in healthy young adults (MacDougall and Moore, 2005; McAuley et al., 2006; Bove et al., 2009; Avanzino et al., 2015; Lagravinese et al., 2017). Thus, this tempo is hinged on the motor repertoire of individuals even in those subjects who do not show peculiar timing abilities. This might explain the absence of differences in motor strategies observed at 2 Hz between TP and CTRL.

Lastly, no difference in movement frequency was found between the two groups; both TP and CTRL succeeded in reproducing the rate imposed by the metronome (as shown by the mean movement frequency values). Therefore, for both supra-second (0.5 Hz) and sub-second (2 Hz) ISIs, the higher TD values observed in TP were compensated by the higher ITI values produced by the CTRL, explaining why the movement frequency values were similar between groups.

### Spatial Accuracy in Bimanual Coordination Test

The percentage of correct sequences did not differ between groups in the bimanual coordination test. Indeed, in both groups, the following findings were obtained: (1) the performance with the RH was more accurate than that of the left one, and (2) the performance at 0.5 Hz was more accurate than that at 2 Hz.

Concerning the higher spatial accuracy obtained with the RH, one should consider that the participants from both groups were right-handed. The significant effect of RATE might find an explanation in the well-known trade-off between movement spatial accuracy and movement speed. Originally as described by Fitts (1954) in relation to voluntary goal-directed movement, the speed–accuracy trade-off law was shown to be valid for different kinds of movement, including finger opposition (Lachnit and Pieper, 1990). In the present study, speed–accuracy trade-off might explain the higher spatial accuracy observed during slow movement (0.5 Hz) in comparison with the lower accuracy in the fast movement (2 Hz); in other words, in order to fulfill the task requiring to move in sync to the metronome, participants were less accurate when moving faster.

### Movement Strategy in Movement Lateralization Test

Both TD and ITI values were longer at 0.5 Hz than at 2 Hz as expected due to the timing constraint imposed by the tasks. In fact, as was observed in the bimanual coordination test, when the time interval between one beat and the following was shorter (i.e., 2 Hz), with the aim to increase movement velocity, the time devoted to both TD and ITI decreased.

Although TD mean values of TP at 0.5 Hz (mean values of SHT and BT) (340.81 ± 28.31 ms) were numerically higher than those of CTRL (290.29 ± 26.99 ms), no significant difference appeared between the two groups.

### Motor Expertise Influences Spatial Accuracy in Movement Lateralization Test

Differences between groups appeared in the movement lateralization test, namely when considering the performance of the right dominant hand during SHT and BT. Only in TP group, and only at 0.5 Hz, performance with the RH was more accurate in the BT than the SHT. Consequently, TP were more accurate than CTRL when performing BTs at 0.5 Hz.

For a sport like tennis, which is generally characterized by a highly asymmetrical training, a more accurate performance with the RH in the BT with respect to the SHT was not to be expected *a priori*. However, this “gain of function” in terms of spatial accuracy of RH performance when performing fine manual sequences bimanually as opposed to a single-hand modality may be put in relation to the ability that TP develop in executing a BT, with a particular reference to the backhand stroke. Indeed, it is worthy to note that all the athletes recruited for this study performed the backhand stroke with two hands. Therefore, they were also highly trained to perform bimanual motor tasks. From a neurophysiological point of view, the corpus callosum is the primary commissural region of the neocortices, consisting of white matter tracts that connect the left and right cerebral hemispheres (Tomasch, 1954). This structure enables sensory, motor, and cognitive integration between the hemispheres (Gazzaniga, 1995). The existing literature tends to support the hypothesis that in people who develop bimanual abilities, such as musicians, the training can induce changes in cross-hemispheric connections, with significant differences in various regions of the corpus callosum in expert vs. nonexpert (Moore et al., 2014). For this reason, in TP, the existence of a similar phenomenon that would be responsible of higher spatial accuracy during the BT might be speculated. Furthermore, a recent review showed that exercise, and in turn, enhanced cardiorespiratory fitness and is associated with structural and functional outcomes of the corpus callosum, in particular in those portions connecting the prefrontal cortices (Loprinzi et al., 2020), namely regions involved in supra-second timing abilities. Tennis can be classified as a mainly anaerobic activity requiring, however, high level of aerobic conditioning to avoid fatigue and aid in recovery, thus promoting continuous success in professional tennis (König et al., 2001; Kovacs, 2006). Therefore, the aerobic fitness of TP might have promoted changes in the prefrontal portion of the corpus callosum, responsible for the improved bimanual ability in the supra-second task here observed.

Interestingly, the difference in accuracy between RH performance in BT and SHT in TP was specific for 0.5 Hz. Supra-second explicit timing tasks (i.e., 0.5 Hz—ISI 2,000 ms) might be ascribed to the cognitively controlled timing system that involves a cortical–subcortical network, encompassing the cerebellum, basal ganglia, and prefrontal and parietal cortices (Lewis and Miall, 2003; Coull and Nobre, 2008; Breska and Ivry, 2016). Both the prefrontal and posterior parietal cortices are involved in intermanual transfer processes, as deducible from studies in both healthy subjects (Garbarini et al., 2014) and in patients with brain damage (Garbarini et al., 2012). Prefrontal cortex has been demonstrated to be an important structure for executive functions including decision-making and anticipations (Funahashi and Andreau, 2013), the efficiency of which varies with the level of motor expertise also in sport contexts (Williams and Jackson, 2019). For these reasons, one might speculate that the better motor performance of TP with respect to CTRL in the BT performed at 0.5 Hz was due to a stronger efficiency of prefrontal-parietal network following practice-dependent plasticity in these athletes.

The speed–accuracy trade-off mechanism previously described can explain the experimental observation that in the BT, in both groups, movement spatial accuracy at 0.5 Hz was significantly higher than those at 2 Hz. Finally, no differences between SHT and BT and between the two groups were observed when the task was performed at 2 Hz, possibly as a result of the correspondence between 2 Hz and spontaneous movement tempo in healthy adults (Bove et al., 2009; Bisio et al., 2015; Lagravinese et al., 2017).

### Limitations of the Study and Future Works

The synchronization paradigm applied here to test the influence of motor expertise on motor performance during timing tasks succeeded in showing differences in kinematics and spatial accuracy between TP and CTRL groups, but failed to reveal a difference in temporal accuracy. One possible explanation is that the synchronization paradigm was not the most suitable to unveil this difference. In fact, studies showing significant differences between groups on synchronization error used the synchronization–continuation paradigm and computed the synchronization error in the continuation phase (Martino et al., 2015, 2019). In order to specifically assess the role of expertise in tennis on timing accuracy, future studies might thus apply the synchronization–continuation paradigm error.

In the present study, the influence that anthropometrical characteristics could have in motor performance was not specifically tested. Although scientific literature on this topic suggests that abilities in motor timing tasks are predominately driven by central processes (Lewis and Miall, 2003; Coull and Nobre, 2008), at present it cannot be possible to definitely rule out their involvement in these results.

Lastly, this study focused on young TP. One might assume that young athletes are still developing timing abilities, but did not reach their best. For that reason, it would be interesting to use the methodology applied in this study to monitor the improvements in the timing abilities of athletes during training.

## Conclusion

This study showed that the motor timing abilities evaluated by a finger-opposition movement task in synch to a metronome paced at different rates differed between TP and CTRL. In particular, when they were involved in the bimanual coordination test, at supra-seconds ISI, motor experience in tennis influenced the motor strategy, leading TP to devote more time to elaborate the input signal and prepare the motor response than CTRL. Differently, in the movement lateralization test, the motor experience influenced spatial accuracy; at supra-second ISI, the percentage of correct sequences of TP was significantly higher than that of CTRL when the task was performed bimanually. It has to be noted that since tennis mainly involves arm and not hand movements, the present differences appear to be related to a high-level motor timing ability, not specific to the trained body part or movement.

## Data Availability Statement

The raw data supporting the conclusions of this article will be made available by the authors, without undue reservation.

## Ethics Statement

The studies involving human participants were reviewed and approved by Comitato Etico per la Ricerca di Ateneo -University of Genoa (no. 2021.31). Written informed consent to participate in this study was provided by the participants' legal guardian/next of kin.

## Author Contributions

AB, EF, and PR contributed conception, design of the study, and critically discussed the results. GC and VF performed the experimental study. AB, EP, and LA organized the database and performed the statistical analysis. EF and AB wrote the first draft of the manuscript. EP, LA, and PR wrote sections of the manuscript. All authors contributed to manuscript revision, read, and approved the submitted version.

## Conflict of Interest

The authors declare that the research was conducted in the absence of any commercial or financial relationships that could be construed as a potential conflict of interest.
